# Experimental Study on Full-Scale Beams Made by Reinforced Alkali Activated Concrete Undergoing Flexure

**DOI:** 10.3390/ma9090739

**Published:** 2016-08-30

**Authors:** Linda Monfardini, Fausto Minelli

**Affiliations:** DICATAM—Department of Civil Engineering, Architecture, Environment, Land Planning and Mathematics, University of Brescia, 25123 Brescia, Italy; fausto.minelli@unibs.it

**Keywords:** alkali activated concrete, fly ash, geopolymer concrete, material characterization, structural characterization

## Abstract

Alkali Activated Concrete (AAC) is an alternative kind of concrete that uses fly ash as a total replacement of Portland cement. Fly ash combined with alkaline solution and cured at high temperature reacts to form a binder. Four point bending tests on two full scale beams made with AAC are described in this paper. Companion small material specimens were also casted with the aim of properly characterizing this new tailored material. The beam’s length was 5000 mm and the cross section was 200 mm × 300 mm. The AAC consisted of fly ash, water, sand 0–4 mm and coarse aggregate 6–10 mm; and the alkaline solution consisted of sodium hydroxide mixed with sodium silicate. No cement was utilized. The maximum aggregate size was 10 mm; fly ash was type F, containing a maximum calcium content of 2%. After a rest period of two days, the beam was cured at 60 °C for 24 h. Data collected and critically discussed included beam deflection, crack patterns, compressive and flexural strength and elastic modulus. Results show how AAC behavior is comparable with Ordinary Portland Cement (OPC) based materials. Nonlinear numerical analyses are finally reported, promoting a better understanding of the structural response.

## 1. Introduction

Concrete is an essential material in the building industry with a global consumption estimated around 25 billion tons every year [1]; it is considered one of the most widely used materials in the world. The production process of concrete, however, has a significant impact on global warming; this is especially due to the use of Ordinary Portland Cement (OPC), the main component of concrete. The cement industry is in fact responsible for about 6%–7% of CO_2_ emissions worldwide [2], which is especially due to fossil fuel used during manufacturing processes that emit greenhouses gases. It has been estimated that the production of one ton of cement releases about one ton of CO_2_ [3] and, moreover, OPC production uses large amounts of raw materials, especially limestone.

The need for environmentally friendly building materials for sustainable development is now a major environmental issue in the construction industry; over the last decades, there has been a remarkable development of alkali activated materials as an alternative kind of concrete that uses an aluminosilicate powder as a total replacement of cement. Alkali activated cements are synthetized from powders rich in alumina and silica which are soluble in highly alkaline solutions. The combination of an aluminosilicate source and alkaline solution react to form a binder, playing a similar role of cement combined with water in the ordinary concrete. The aluminosilicate base that could be activated through the alkaline solution could be a metakaolin or industrial byproduct material such as fly ash and ground-granulated blast furnace slags. The remaining mixture ingredients are ordinary coarse and fine aggregates and eventually extra water to improve workability. 

Early research about alkali activated materials began in the 1940s with Purdon’s [4] studies, in which activation through sodium hydroxide solution of blast furnace slag was analyzed. Later, in the 1959, Gluchovskij [5] investigated the synthesis of new binding materials through the reaction of aluminosilicate raw materials with alkaline compounds. In 1976, Davidovits [6] defined the term “*geopolymer*” to classify the geosynthesis that produces inorganic polymeric materials used for a number of industrial applications. 

Regarding the mechanical characterization of AAC there is a significant amount of literature. Different authors [7,8] demonstrated that the combination of different materials composing AAC is fundamental for compressive strength development; similarly to OPC, a main role is played by the equivalent *water*/*geopolymer solids* ratio; compressive strength increases when this ratio decreases. An increase in sodium hydroxide molarity is favorable for strength. Compressive strength investigated reached values close to 80 MPa.

Curing at high temperature allows AAC to achieve good mechanical performance in a short period of time. The curing process takes place after mold removal, AAC is heated up to 60 °C or more, a rapid polymerization occurs and the compressive strength slightly increases over time, showing almost the same values between 7 and 28 days [9,10]. On the other hand, under ambient curing, an increase of compressive strength of about 10–15 MPa between 7 and 28 days occurs due to protraction of polymerization reaction [11].

Elastic modulus has been investigated by different authors [9,10,11,12] and compared with different national standards. In general, experimental elastic modulus has found to be lower than that predicted by standard codes, depending on the coarse aggregate used in the mixture.

Experimental data on the complete stress–strain curve of AAC are scarce in literature; Hardjito et al. [9] found a good fitting between experimental data and analytical curves for OPC concrete. Sarker [13] modified the post-peak existing stress–strain model originally proposed by Popovics [14] for OPC concrete to predict AAC behavior, evidencing a slightly more brittle post-peak behavior. 

As found by different authors [9,11], tensile and flexural strength present a better performance than that obtained through standards for OPC-based concretes. Sarker [15] investigated the bond between AAC and steel rebar: AAC presents a better bond strength because of higher splitting tensile strength. In addition, the interfacial transition zone (ITZ) between aggregate and paste is denser compared to the one in OPC. Fernandez provided similar results [10].

Regarding the addition of superplasticizers, there are different theories in literature on their efficiency: some authors [16] experimented an improvement in workability, others did not find any enhancement [17,18]. Nematollahi [19] concluded that efficiency of superplasticizer admixtures on the workability and strength of fly ash-based mixtures directly depend on the type of alkaline activator and the chemistry of the superplasticizer.

Data about structural behavior of AAC full-scale elements are scarce in literature: Yost et al. [12,20] investigated the behavior in shear of reinforced AAC beams: data collected included load-displacement plot, analysis of strain and crack patterns that showed a behavior very similar to OPC-based structural elements. Similar results have been found by Sumajow et al. [21] who tested AAC beams; experimental data were in agreement with Australian standards for concrete structures. Sumajow et al. [22,23] focused on the behavior of AAC columns loaded with an eccentric axial load; the failure was due, as expected, to AAC crushing. Results showed good correlation with American Concrete Institute Building Code ACI 318-02 [24] and the Australian Standard AS3600 [25].

NG et al. [26] investigated the shear behavior of AAC beams reinforced with steel fibers in various volumetric dosages. They found an increase in the final shear strength and in the cracking load with increasing fiber volume. 

The project described herein, which comprises two full-scale tests on beams under flexure and a set of tests for material characterization, was aimed at evaluating the AAC’s mechanical properties, under both a structural and material point of view, in order to foresee its utilization in the construction field, especially in structural applications related to both light (duct elements, manholes, etc.) and heavy (beams, columns, roof elements, etc.) prefabrication.

## 2. Materials and Methods

### 2.1. Specimens Geometry

**Figure 1:**
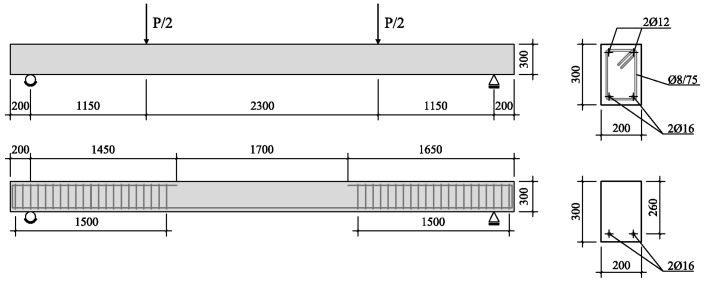
Sample details for full-scale beams, dimensions in mm.

Two full-scale beams (AAC1-HC and AAC2-HC) entirely made by AAC were produced and tested under a four point loading system; beams had an overall length of 5000 mm, a span length of 4600 mm and a cross section of 200 mm × 300 mm. Specimens were longitudinally reinforced with two Ф16 mm (*A_s,long_* = 402 mm^2^) bars in tension; as for OPC design, flexural longitudinal reinforcement was designed by imposing a minimum steel percentage ρ*_s_* (0.77%) that provides good ductility to the structural element and promotes rebar yielding. In the middle portion of the element (in the flexural span between the two point loads), no stirrups and reinforced bars in compression were provided in a way to have a flexural collapse, depending only on the concrete crushing. In the remaining portions of the beam, two Ф12 mm bars were provided in the compression zone to hold stirrups in position; closed stirrups (Ф8 mm@75 mm) were supplied to avoid brittle collapse due to shear. The resulting transversal steel ratio ρ*_v_* is 0.67% and the clear concrete cover is 24 mm. Figure 1 summarizes the main characteristics of the structural element. 

For each beam, companion specimens were casted for compression and Modulus of Rupture (MOR) material tests. Cylinder specimens (*D* = 100 mm, *H* = 200 mm) were tested in compression to derive Young’s modulus [27] and the complete stress-strain behavior; in addition, compressive strength was also calculated over time through cube specimens (*L* = 100 mm). As far as MOR is concerned, beam notched specimens (150 mm × 150 mm × 600 mm) were tested according to EN14651-5 [28].

### 2.2. Material Properties and Curing Conditions

The AAC beams and companion specimens were fabricated at the Material Laboratory of the University of Brescia. A single mix design was used for both castings. Coarse aggregate 6–10 mm (1201 kg/m^3^), sand 0–4 mm (647 kg/m^3^) and class F fly ash (408 kg/m^3^) were mixed with 8 M sodium hydroxide (41 kg/m^3^), sodium silicate (103 kg/m^3^) and extra water (35 kg/m^3^); the resulting volumetric mass density (2435 kg/m^3^) is comparable to ordinary concrete. The mixture presents an alkaline solution/fly ash ratio of 0.35 and alkaline solution + extra water/fly ash ratio of 0.42. In order to fit structural applications, Davidovits [29] suggested a Si/Al ratio equal to 2: the resulting Si/Al ratio of the mixture is equal to 2.26 considering both contribution from fly ash and sodium silicate. 

Class F fly ash was provided by a local supplier: the typical chemical composition is shown in Table 1. A low content (<5%) of CaO was required in a way to avoid fast setting and consequently to have better workability conditions; moreover, Loss of Inhibition (LOI) should be less than 5% not to inhibit the alkali reaction [30]. No superplasticizer was used in the final mixture since it was found from several pilot mixtures that no contribution in terms of workability was achieved with many commercial products for OPC.

Alkaline solution, composed of sodium hydroxide in solution (8 M) and sodium silicate (Weight Ratio WR = 1.99), was prepared a few hours before the pouring and left at room temperature until the use; coarse aggregates with a maximum size of 10 mm were used not to reduce workability. Aggregates humidity was calculated in a way to adjust extra water amount during casting. Due to the large quantity used in both castings, few kilograms of aggregates were utilized. The water content obtained from the drying process was considered representative for the overall humidity definition. 

The material was produced through a planetary mixer: solid parts (fly ash + aggregates) were added first and, after 4 min of mixing, remaining liquid parts (alkaline solution + extra water for workability) were incorporated, and another 4–5 min of mixing followed.

This mix design currently used was the result of several previous pilot experiments carried out to obtain a normal strength AAC with a good workability. To define the optimal mix design, different curing parameters have been investigated, as summarized in Table 2. Parameter values have been chosen according to the greatest compressive strength they determined at 7 days. 

AAC was poured into wood formworks; cylinder formworks were the only ones made of steel; their internal surface was covered by greaseproof paper in order to simplify removal. Because of the high consistency of the concrete, good compaction was achieved using a rod and a vibrating needle. Workability was evaluated after cast, using an Abram’s cone; concrete was in class S1 for AAC1-HC batch (slump: 40 mm) and class S2 for AAC2-HC (slump: 70 mm). 

**Figure 2:**
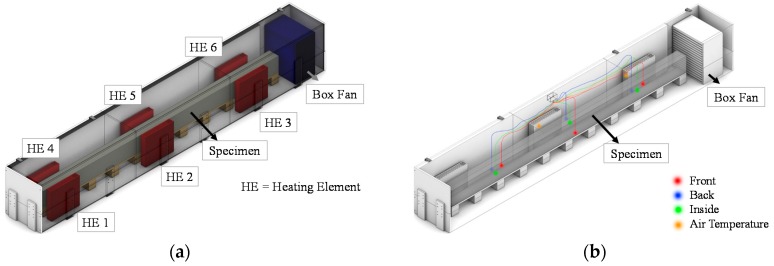
Hand-made curing chamber: position of heating elements (**a**); and monitoring instrumentation (**b**).

After a rest period of 48 h, beams and companion specimens were covered with two layers of plastic and a saturated cotton sheet in the middle of them; covering layers had the aim of retaining moisture during the curing. Curing at an elevated temperature followed: specimens were placed in a hand-made climatic chamber (Figure 2) for 24 h at 60 °C. The source of heat consisted of three radiators placed on each side of the beam length; a fan was also provided with the aim of uniformly heating the chamber, whereas temperature was monitored through sensors placed both inside and outside the volume of the beams.

Regarding reinforcement, typical Italian B450C steel was used for the entire steel reinforcement cage; Ф16 mm rebar had measured yield (*f_y_*) and ultimate (*f_u_*) strengths of 546 and 649 MPa, respectively; properties were evaluated according to EN-15630-1 [31], testing two 600 mm long coupons.

### 2.3. Test Setup and Investigation

**Figure 3:**
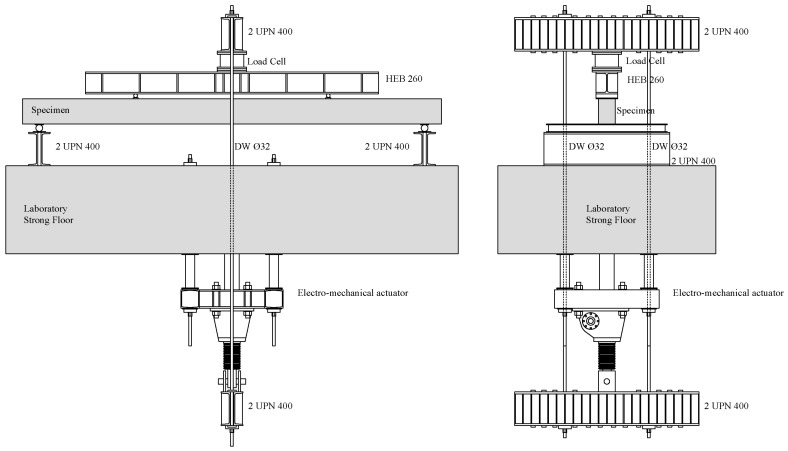
Scheme of loading system adopted.

Beam AAC1-HC and Beam AAC2-HC were tested under flexure, respectively, at 31 and 8 days after casting. A displacement controlled test was guaranteed allowing for a suitable and stable control during critical steps, such as in the case of abrupt cracking phenomena or load drops. The applied load was measured by a single load cell. In the setup used for testing under a four point loading system (Figure 3), both the two point loads and the two supports were modeled though steel profiles; they were continuous on the entire specimen width. In order to avoid load concentration and possible local failure, a suitable steel plate on a mortar bed was disposed at each loading point.

**Figure 4:**
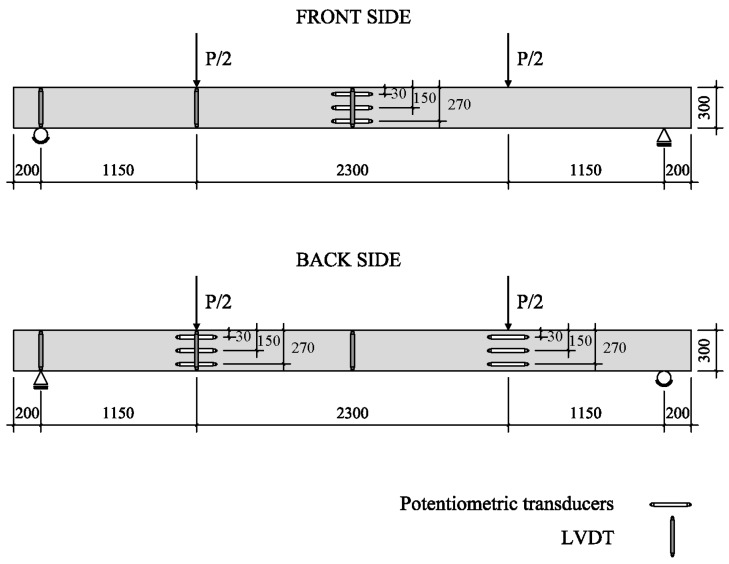
Instrumentation position. Potentiometers (±125 mm range), LVDTs at midspan (±100 mm range), under load point (±50 mm range) and at supports (±20 mm range).

Linear Variable Differential Transformers (LVDTs) were utilized for measuring deflections at mid-span (front and back side), under the load points and at supports. Potentiometric transducers were adopted for measuring the flexural strains, hence the curvature; three were located at front mid-span, whereas the other two groups (of three each) were positioned in the back side under the two concentrated loads (Figure 4). After preliminary elastic cycles, all tests were conducted by loading the beams under monotonically increasing screw displacement up to failure. The screw rate was set at 1 mm/min in the initial stage, reduced to 0.75 mm/min from 80 kN. Load applied during elastic cycles was lower than the first cracking load; elastic cycles were meant for the instrumentation final control and calibration. The test was stopped at different load values in order to track the development of crack pattern.

Beams and cylinders were tested at 28 days in flexure and in compression respectively; flexural tensile stress vs. Crack Mouth Opening Displacement (CMOD) curves were obtained according to EN 14651-5 [28], which requires that three point bending tests on notched specimens be performed under strain control. Even though this test is meant firstly for characterizing fiber reinforced concrete (FRC) materials, it was considered useful for investigating the post-peak regime of AAC materials. 

Cylinders were tested to investigate both elastic modulus [27] and stress–strain relationship. In order to obtain constitutive law, the same instrumentation setup of elastic modulus was used. Three LVDTs were used to measure the axial deformation of the concrete cylinders. This test was run in stroke control using a universal closed-loop Instron machine. Cubic compressive strength was measured 7, 14 and 28 days after casting.

## 3. Results and Discussion

### 3.1. Modulus of Elasticity and Compression Tests

**Figure 5:**
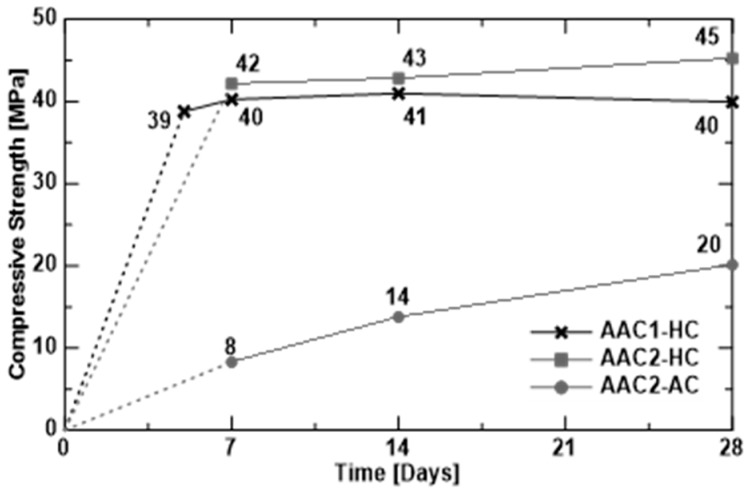
Compressive cubic strength versus time (HC = Heat Curing, and AC = Ambient curing).

Figure 5 shows the development of the cubic compressive strength over time; because of the heat curing (HC), compressive strength did not have an increase in time but it maintained almost constant. The mean cubic compressive strength measured on 28th day was 40 MPa (COV 3.67% on three specimens) for AAC1-HC and 45 MPa (COV 5.46% on three specimens) for AAC2-HC.

Some cubes from AAC2 casting were cured at ambient condition (AC). Unlike the previous case, they did not show a constant value of compressive strength: they act like OPC concrete, showing a gradual increase over time; however, compressive strength after 28 days was almost half of that obtained from heat curing (HC) conditions. As reported in the literature [32], the compressive strength of ambient-cured specimens increases with age, depending on the average ambient temperature: the higher the ambient average temperature, the higher the strength.

**Figure 6:**
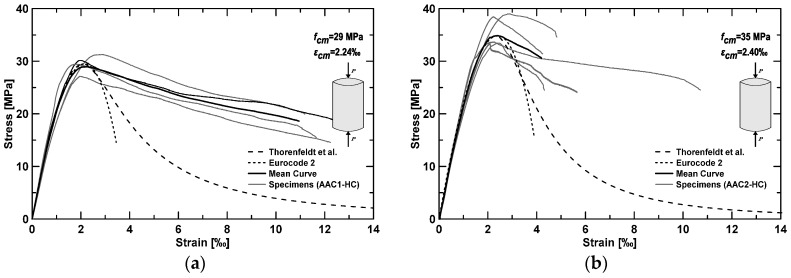
Stress–strain relationship in compression for: AAC1-HC (**a**); and AAC2-HC (**b**).

The corresponding cylinder compressive strength was analytically determined, according to OPC concrete, using the following relation [33]:

(1) $$f_{cm}=0.83\;R_{cm}$$

where *R_cm_* represents the average cubic compressive strength on 28th day; results show a cylinder compressive strength equal to 33.2 and 37.3 MPa, respectively, for AAC1-HC and AAC2-HC specimens. Compression tests on cylinders (Figure 6) provided slightly lower cylinder compressive strength (29 and 35 MPa, COV 5.21% and 7.26% on four and five specimens, respectively, for AAC1-HC and AAC2-HC) with a mean value of 32 MPa. The scatter between experimental and analytical values from Equation (1) ranges between 6% and 12%.

Young’s modulus, determined from cylinder specimens according to [27], was equal to 24 GPa for AAC1-HC (COV 8.32% on five specimens) and 25.2 GPa (COV 5.19% on six specimens) for AAC2-HC, resulting in a mean value of 24.6 GPa.

For OPC concrete, Eurocode 2 (EC2) [34] recommends the following relation:

(2) $$E_{cm}=22000{\left(\frac{f_{cm}}{10}\right)}^{0.3}\;(MPa),$$

where *f_cm_* represents the mean cylindrical strength on 28th day.

Using the above equation for the range of *f_cm_* experimentally obtained (29 and 35 MPa), an analytical value of *E_cm_* comprised between 30.2 and 32 GPa would be determined. Therefore, the experimental Young’s modulus is in both cases about 80% of that predicted by EC2 [34] for OPC concretes, which might be due to the lower value of the maximum aggregate size (10 mm) herein utilized.

The experimental stress–strain relationships in compression are illustrated, for both materials, in Figure 6. Experimental results have been compared with the following two analytical formulas for OPC concretes: Thorenfeldt et al. [35] and the current EC2 [34]. The first relationship is as follows:

(3) $$\sigma_{c}=f_{cm}\cdot \frac{\varepsilon_{c}}{\varepsilon_{cm}}\cdot \frac{n}{n-1+{\left(\frac{\varepsilon_{c}}{\varepsilon_{cm}}\right)}^{nk}}$$

where

*f_cm_* = peak stress;
ε*_cm_* = strain corresponding at peak stress;
*n* = 0.8 + (*f_cm_*/17);
*k* = 0.67 + (*f_cm_*/62), if ε*_c_*/ε*_cm_* > 1; and
*k* = 1, if ε*_c_*/ε*_cm_* ≤ 1;

while the EC2 [35] formulation is:

(4) $$\sigma_{c}=f_{cm}\cdot \frac{k\eta -\eta^{2}}{1+(k-2)\eta }$$

where

*f_cm_* = peak stress;
η = ε_c_/ε_cm_;
ε*_cm_* = strain corresponding at peak stress; and
*k* = 1.05 *E_cm_*|ε*_cm_*|/*f_cm_*.

Values included in the previous analytical formulas refer to experimental results. Analytical formulas fit well with the experiments only in the pre-peak phase, whereas, in the post-peak phase, AAC shows a much more ductile behavior. This is in clear contrast with a previously published study [13], which showed a similar trend (slightly more brittle) compared to OPC.

### 3.2. Material Flexural Behavior (MOR Tests)

**Figure 7:**
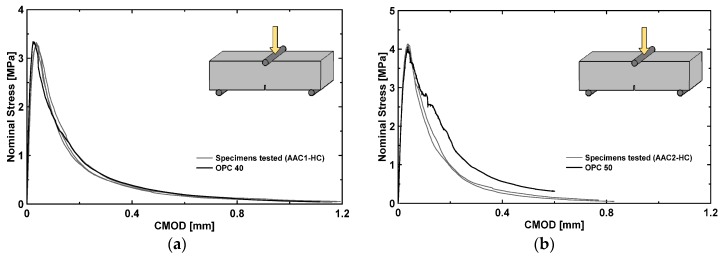
Tensile stress–CMOD relationship for: AAC1-HC (**a**); and AAC2-HC (**b**).

Flexural tensile stress–CMOD relationship for both materials is shown in Figure 7; two or more AAC samples for each casting have been tested. In this picture, a classical OPC concrete beam response (having similar peak strength) is also provided for comparison: no significant differences in the behavior of the two tested materials were observed. Unlike the behavior in compression, AAC materials and OPC concretes are both extremely brittle in tension, showing a fast decrease in strength, with residual stresses lower than 0.3 MPa for CMOD values greater than 0.6 mm.

### 3.3. Structural Response

**Figure 8:**
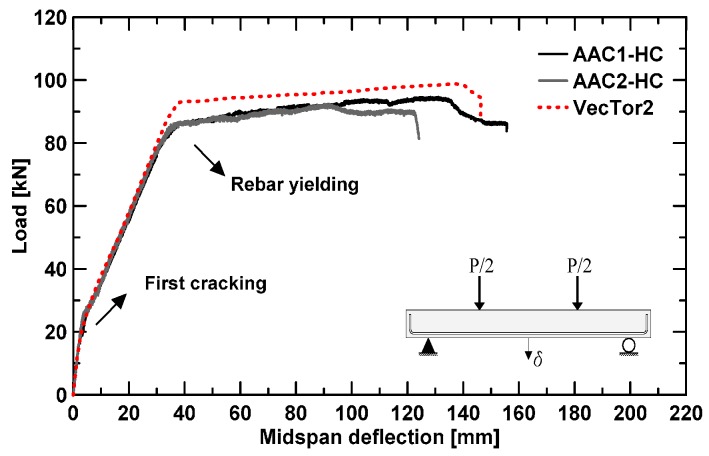
Experimental and numerical load–deflection curves.

The load–midspan deflection curves of AAC1-HC and AAC2-HC full-scale beams are reported in Figure 8. AAC beams act like OPC showing an elastic range (uncracked stage), followed by a cracked-elastic stage up to a clear yielding of rebar.

Mid-span deflections have been monitored by two LVDTs, located in the front and in the back of the beam at midspan (Figure 4 for details); for the deflection calculation, the influence of support displacement was also taken into account.

Despite the different Young’s modulus and testing time (AAC1-HC has been tested 31 days after pouring, whereas AAC2-HC after eight days only), beams behavior is almost identical; experimental curves fit very well especially in the pre-yielding phase. Table 3 summarizes all the main experimental results for both beams.

A prediction of both ultimate load *P_u_* and flexural moment *M_u_* was provided using the following formula that assumes the rebar yielding, as experimentally observed:

(5) $$M_{u}=0.9d\cdot A_{s}\cdot f_{y}\;=\;51\text{ kNm},$$

(6) $$P_{u}=\frac{2\cdot M_{u}}{a}\;=\;89\text{ kN},$$

where

*d* = effective depth (260 mm);
*A_s_* = longitudinal reinforcement (402 mm^2^);
*f_y_* = steel yield strengths (546 MPa); and
*a* = shear span (1150 mm).

**Figure 9:**
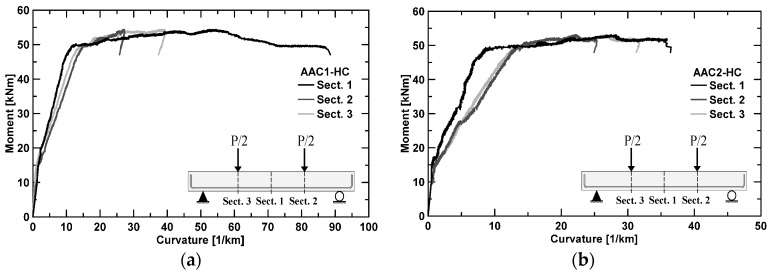
Moment-Curvature curves for: AAC1-HC (**a**); and AAC2-HC (**b**), at different sections.

**Figure 10:**
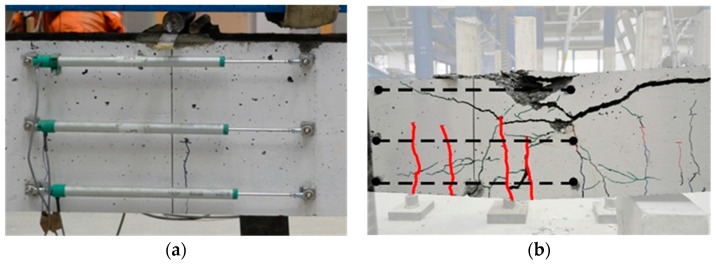
Example of potentiometers position before the test (**a**); and cracks developed along instrumentation (**b**).

Figure 9 illustrates the experimental local moment–curvature curves at different cross sections obtained for the two structural elements; the moment was directly derived by the applied load and curvature from potentiometers readings. For the curvature calculations, only the top and bottom potentiometers were considered. Again, moment–curvature development has the typical trend of OPC materials; the difference in the flexural stiffness *EJ* is due to the number of cracks developed along the instrumentation position (Figure 10); a greater number of cracks produced a decrease in the stiffness.

Both the load–midspan deflection and moment–curvature curves were obtained considering the self weight of the beam and the loading system, the latter weighting 16 kN. 

Ultimate load values obtained from the test are 95 and 92 kN, respectively, for AAC1-HC and AAC2-HC. Therefore, experimental data slightly exceed the prediction by 6.7% (AAC1-HC) and 3.4% (AAC2-HC). The moment at yielding is for both beam around 50 kNm, as well predicted by Equation (5).

Ductility values, calculated as a ratio between ultimate and yielding displacement, are, respectively, equal to 4.22 and 3.24 for AAC1-HC and AAC2-HC, as also shown in Table 3.

**Figure 11:**
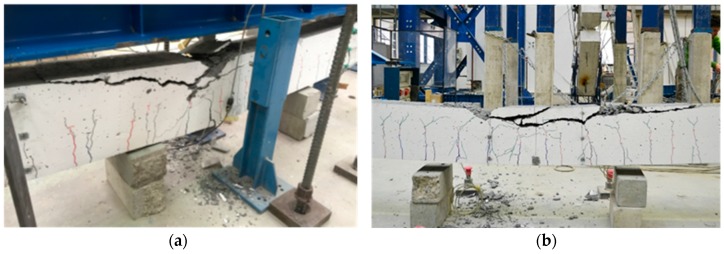
Concrete crushing, with yielding of rebar, for: AAC1-HC (**a**); and AAC2-HC (**b**).

As expected, even though well beyond rebar yielding, collapse was due to concrete crushing at midspan, as shown in Figure 11.

### 3.4. Numerical Analyses

Experimental load–midspan deflection curves are compared with an analytical curve obtained through the nonlinear finite element analysis program VecTor2 (VT2), developed by Professor Vecchio at the University of Toronto [36] and based on the Modified Compression Field Theory [37]. Since the mixture design is the same for both beams and experimental curves are almost identical, only one numerical curve was derived by averaging all material input data derived from material characterization tests.

Input data for VT2 for both concrete and steel materials are illustrated in Table 4. A 2D plane stress finite element model was adopted; moreover, the finite element mesh was characterized by having four-noded elements (around 20 mm × 20 mm in size) with uniform thickness (200 mm). In order to obtain consistent numerical predictions (no mesh sensitivity), at least 15 elements were modeled along the depth of the specimens after a pilot sensitivity study. Reinforcing bars were defined as an elasto-plastic material, with hardening, by means of a multi-linear stress–strain curve representing the actual response of several bars tested. Rebar were modeled as truss reinforcement, perfectly bonded to the surrounding concrete.

The crack spacing was numerically evaluated by the classical formulation, implemented in the program VT2 and adapted from Model Code 1978 [38].

**Figure 12:**
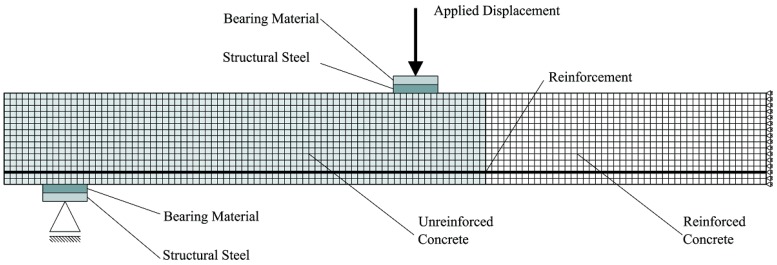
Finite element model adopted.

Figure 12 depicts a typical mesh constructed for the numerical analyses.

In order to avoid unrealistic punching (or local) failure and numerical instability due to load concentration, point load and supports were simulated as nodal loads acting on a steel plate having a longitudinal dimension of six finite elements, connected to the concrete elements through a bearing material (Figure 12), for simulating mortar beds that were experimentally provided between specimen and any support/loading plate.

The obtained analytical curve fits quite well with the experimental data, especially in the uncracked and cracked-elastic stage, with a good modeling of the phenomena related to the post-cracking stiffness, cracking formation and progression, tension stiffening effect and others. The modeling, on the other hand, tends to slightly overestimate the load at yielding and the maximum capacity by about 7.5%–8.6% and 4%–7.1%, respectively.

Main load and displacement values are summarized in Table 3, where a comparison with experimental results is also provided.

Based on this preliminary study, it can be said that the set of nonlinear constitutive models, typical of classical reinforced concrete elements, are also able to satisfactorily model alkali activated reinforced concrete beams, hence envisaging a safe utilization of these available tools for a better understanding of structural implications of these new materials.

### 3.5. Crack Pattern

**Figure 13:**
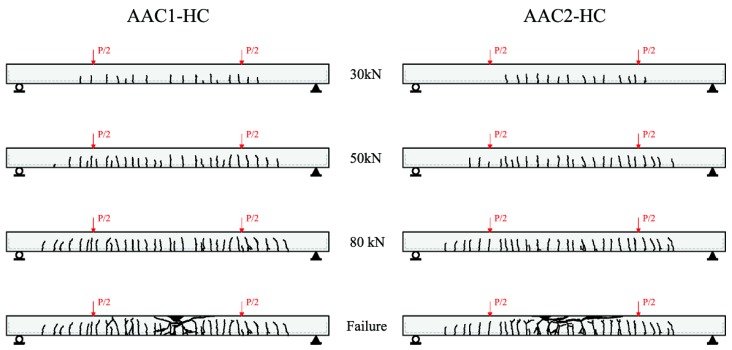
Crack pattern at different load levels (front side) of AAC1-HC and AAC2-HC beams.

Figure 13 shows crack patterns for both beams at four different load levels: 30, 50, and 80 kN, and at collapse. The three values refer to a loading condition just after the first cracking (30 kN), at a typical service condition (50 kN) in which the cracking phenomenon is stabilized, and just before the rebar yielding (80 kN). As expected, flexure cracks initiated in the pure bending zone (flexural span). As the load increased, existing cracks propagated and new cracks developed along the span. The crack patterns observed for AAC beams were similar to those reported in the literature for reinforced OPC beams.

Cracks were able to penetrate into the member depth even for low loads: mean depth of cracks for the first load level (30 kN) was comprised between 10 and 15 cm. In the following load steps, pre-existing cracks further developed and new cracks formed (none after 80 kN). Some of these cracks, especially at mid-span, opened widely near failure. After rebar yielding, minor shear cracks also started to develop, as shown in Figure 13.

Mean crack spacing was experimentally evaluated and compared with CEB-FIP Model Code 1978 [38] formula, developed for OPC structural elements:

(7) $$S_{m}=2\left(c+\frac{s}{10}\right)+k_{1}\cdot k_{2}\cdot \frac{Ф}{\rho_{eff}}$$

where

*c* = concrete clear cover;
*s* = distance between longitudinal reinforcement bars;
*k*_1_ = coefficient regarding bond between bars and concrete (*k*_1_ = 0.4)
*k*_2_ = coefficient regarding stress distribution in the cross section (*k*_2_ = 0.125);
Ф = longitudinal reinforcement bars diameter; and
ρ*_eff_* = effective reinforcement ratio.

The previous formula in Equation (7) predicts a crack spacing of 115 mm, which is almost coincident with the mean experimental crack spacing, varying from 102 to 113 mm (see Table 3). The cracking formation and development are therefore rather similar to well acknowledged classical reinforced concrete beams.

## 4. Conclusions

The experimental results of two flexure tests on full-scale beams made of AAC were presented and discussed in this paper, focusing on the material characterization, on the structural response and on a preliminary analytical investigation.

Based on the results of this experimental investigation, the following conclusions can be drawn regarding material characterization:

(1) Due to heat process, no increase in compressive strength over time (between 7 and 28 days) was noticed.
(2) MOR tests, besides the peak strength, pointed out a rather brittle behavior in tension of the AAC herein considered, coincident to OPC.
(3) Experimental Young’s modulus resulted around 20% lower than OPC concrete, which might be due to the use of a 10 mm maximum aggregate size in the mix design.
(4) Constitutive law in compression was found to have a trend similar to analytical formulas proposed for OPC only in the pre-peak response. On the contrary, in the post-peak response, AAC presented rather higher ductility, in contrast with other authors.

About structural behavior:

(5) Despite a difference in testing time, flexural behavior of full-scale beams did not show significant differences in terms of the overall response, uncracked and cracked-elastic parameters, yielding and ultimate load.
(6) The overall behavior under flexure (cracking formation and development included) was seen to be similar to an ordinary reinforced beam made of OPC concrete, showing failure due to concrete crushing after rebar yielding.
(7) The preliminary nonlinear finite element analyses performed, using a set of equations describing the linear and nonlinear phenomena of classical OPC reinforced concrete elements, gave a rather good modeling of the AAC tested beams, promoting the utilization of these already available tools for further analyses able to improve the structural understanding of ACC members.

## Figures and Tables

**Table 1:** Chemical composition of Class F fly ash.

Component	Al_2_O_3_	SiO_2_ *	CaO	Fe_2_O_3_	MgO	K_2_O	Na_2_O	TiO_2_	SO_3_
	(%)	(%)	(%)	(%)	(%)	(%)	(%)	(%)	(%)
**Percent Composition**	28	56	2	5.5	0.2 ÷ 3	0.2 ÷ 2	0.1 ÷ 0.6	0.1 ÷ 1.7	0.2 ÷ 2

* where 40% is composed by reactive silica, representative of pozzolanic potential of fly ash.

**Table 2:** Choice of curing and mixing parameters.

Parameter Investigated	Best Value
Extra water content, kg/m^3^	25–35
Total mixing time, min	8–10
Rest period, h	48
Curing time at 60 °C, h	24

**Table 3:** Comparison between experimental and analytical results.

Results Investigated	AAC1-HC	AAC2-HC	VT2	Comparison AAC1-HC-VT2 (%)	Comparison AAC2-HC-VT2 (%)
Maximum Load, kN	95	92	99	4.0	7.1
Displacement at maximum load, mm	131	90	136	3.7	33.8
Ultimate displacement, mm	156	123	146	−6.8	15.8
Ultimate load, kN	86	88	94	8.5	6.4
Ultimate displacement/span length	1/29	1/37	1/32	−10.3	13.5
Yielding displacement, mm	37	38	38	2.6	0.0
Yielding load, kN	85	86	93	8.6	7.5
Ductility	4.22	3.24	3.84	−9.7	15.8
Mean crack spacing, mm	102	113	-	-	-

**Table 4:** Input data for VT2.

**Unreinforced Concrete Parameters**	
Cylinder compressive Strength *f*’*_c_*	32 MPa
Elastic modulus *E*	24.6 GPa
Maximum aggregate size	10 mm
**Reinforced Concrete Parameters**	
Cylinder compressive strength *f*’*_c_*	32 MPa
Elastic modulus *E*	24.6 GPa
Maximum aggregate size	10 mm
Reinforcement direction	90 deg
Reinforcement ratio	0.67%
Transverse reinforcement yield strength *f_y_*	545 MPa
Transverse reinforcement ultimate strength *f_u_*	649 MPa
Transverse reinforcement elastic modulus *E_s_*	206 GPa
Transverse reinforcement strain hardening ε*_sh_*	7 millistrain
Transverse reinforcement ultimate strain ε*_u_*	100 millistrain
**Longitudinal Steel Reinforcement Parameters**	
Yield Strength *f_y_*	545 MPa
Ultimate Strength *f_u_*	649 MPa
Elastic Modulus *E_s_*	206 GPa
Strain Hardening ε*_sh_*	7 millistrain
Ultimate Strain ε*_u_*	100 millistrain
**Bearing Material Parameters**	
Direction	90° to the horizontal
**Structural Steel Parameters**	
Yield Strength *f_y_*	500 MPa
